# A multicenter prospective trial evaluating fetal bovine dermal graft (Xenform^® ^Matrix) for pelvic reconstructive surgery

**DOI:** 10.1186/1471-2490-10-21

**Published:** 2010-12-13

**Authors:** Howard B Goldstein, Joseph Maccarone, Martin J Naughton, Oscar A Aguirre, Rakesh C Patel

**Affiliations:** 1 Cooper University Hospital, University of Medicine and Dentistry of New Jersey-Robert Wood Johnson Medical School, Camden, New Jersey, USA; 2 St. Mary's Regional Medical Center, Reno, Nevada, USA; 3 Pelvic Specialty Care and Milestone Medical Research, Inc., Englewood, Colorado, USA; 4 Winter Park Urology Associates, Orlando, Florida, USA

## Abstract

**Background:**

A prospective multicenter clinical study was performed to evaluate the safety and efficacy of a bovine dermal graft (Xenform^® ^Matrix, Boston Scientific, Natick, MA, USA) during vaginal reconstructive surgery.

**Methods:**

Forty-five women with ICS stage 2 or higher pelvic organ prolapse (POP) were enrolled at 4 centers. POP-Q, pelvic floor function (PFDI-20), sexual function (PISQ-12), and patient satisfaction tools were used to assess subjects at baseline, and at 2 and 6 weeks, and 3, 6 and 12 months post surgery. The significance of symptom score changes at 6 months and 1 year were determined by the t-test for paired data. Forty-three of the 45 patients completed the 12 month study.

**Results:**

The majority of the subjects had cystocele (98%) and/or rectocele (84%) defects at study entry. At 12 months, 74% of the defects had improved to a stage 0 or 1. Mean PFDI-20 scores improved by 72% (p < 0.001) at 12 months, and PISQ-12 scores were maintained during the follow-up period indicating no decline in sexual function. Three subjects experienced one serious adverse event each; one of the adverse events (constipation) was deemed by the study physician to be unrelated to Xenform^®^. One subject had severe pyelonephritis resulting in dialysis. This subject had a previous history of pyelonephritis, sepsis and acute renal failure. The third subject had a reported recurrent cystocele of moderate severity, possibly related to the device. No graft related erosions or pain lasting more than 30 days were reported. No subjects withdrew due to an adverse event.

**Conclusion:**

This study is the first to investigate the use of Xenform^® ^Matrix in vaginal reconstructive surgery among patients with POP. Significant improvement was maintained at 12 months utilizing both objective and subjective assessment tools, confirming the safety and efficacy of this material in vaginal surgery.

**Trial Registration:**

ClinicalTrials.gov NCT01244165

## Background

Women face an 11% lifetime risk of requiring major surgery for pelvic organ prolapse (POP) [1]. Among the goals of pelvic reconstructive surgery for POP are relief of symptoms, restoration of normal anatomy, maintenance of vaginal capacity for sexual function, and improvement in quality of life. Even though the surgical techniques for repair of POP continue to be refined and improved, the re-operation rate for persistent or recurrent prolapse is relatively high [2]. In an effort to improve long term success rates of POP surgery, surgeons have incorporated various synthetic or biological prosthetic materials in pelvic reconstructive procedures, as is also done in abdominal wall hernia repair.

Graft materials have long been used as adjuncts, with excellent efficacy and safety, and their use may be extended into anterior and posterior vaginal repairs. In this way, grafts are being used to reinforce repairs, or to replace the supportive tissue that is either deficient or absent [3]. Depending on their placement, grafts can correct both central and lateral anterior vaginal wall defects, as well as those involving the rectovaginal septum.

Biological grafts offer potential advantages over the available synthetic patches. These include the capability for in-vivo tissue remodeling and consequent reduction in the rates of extrusion and/or erosion [4]. Limitations include the inherent biological variability of tissue, cost of the material, size constraints related to harvest sites, and lack of long term data [4]. The types of biological grafts used in pelvic reconstructive surgery have included autografts, allografts, and xenografts [5]. Of these, acellular xenografts are attractive because they are readily available with more uniform thickness, and exhibit good biocompatibility. Xenform^® ^Matrix (Boston Scientific Corp., Natick, Massachusetts, USA), is an acellular noncrosslinked collagen-based xenograft derived from fetal bovine dermis, and is designed to provide a sturdy molecular scaffold that is gradually reabsorbed and replaced by native connective tissue. Xenogenic collagen-based graft substitutes have become increasingly popular for dural repair, where a canine duraplasty model has confirmed collagen remodeling with the Durepair^® ^fiber network (Medtronic, St Paul, Minnesota, USA) becoming increasingly populated by fibroblast and a supporting vasculature at 1, 3 and 6 months [6].

The objective of this multicenter trial was to evaluate the safety and effectiveness of Xenform Matrix with respect to restoration of normal anatomy, symptom relief, quality of life, and sexual function over a 1 year period.

## Methods

A prospective cohort of women who received Xenform Matrix during pelvic reconstructive surgery at four US centers from June 2004 to October 2005 was studied. Prior to initiating this trial all four centers received approval from the Institutional Review Boards at their institutions. This trial was conducted in accordance with the principles espoused by the Declaration of Helsinki and all local regulations. All patients provided written informed consent. Xenform Soft Tissue Repair Matrix was developed and manufactured for this study by TEI Biosciences Inc. (Boston, Massachusetts, USA), and was referred to as Cytrix™ in this multicenter clinical trial; this material is now referred to as Xenform^® ^Matrix--a registered trademark of Boston Scientific Corporation (Natick, Massachusetts, USA).

### Eligibility

Women over 18 years of age with International Continence Society (ICS) stage 2 or greater POP were eligible for study entry. Exclusion criteria consisted of dermal graft implantation during prior pelvic surgery; diabetes mellitus; morbid obesity; a pelvic mass; unexplained abnormal vaginal bleeding; infection; coagulopathy; participation in another investigational device or drug study; pregnancy; life expectancy <2 years; hypersensitivity to collagen or bovine materials; history of soft tissue pathology at the intended implant site; impaired wound healing; and any autoimmune connective tissue disease.

### Endpoints

All subjects entered into the study were examined or interviewed over the phone at 2 weeks, 6 weeks, 3 months, 6 months, and 1 year after their surgery. Postoperative symptomatic relief was assessed through the use of a standardized validated instrument: the Pelvic Floor Distress Inventory (PFDI-20) [7]. Postoperative sexual function was assessed through the use of a standardized validated instrument: the Pelvic Organ Prolapse Sexual Questionnaire (PISQ-12) [8]. The PFDI-20 is a psychometrically validated, condition specific questionnaire consisting of three scales addressing POP distress, colorectal and anal distress, and urinary distress. The PFDI-20 has been shown to be reliable and responsive in a comprehensive assessment of the effect of pelvic floor disorders on quality of life [7]. The PISQ-12, developed specifically to evaluate sexual function in women with POP and/or urinary incontinence, has been validated and shown to exhibit moderate to high reliability [8]. Anatomical success was defined as an improvement of greater than 1 ICS stage in POP compared to baseline. Pelvic organ prolapse quantification (POP-Q) is a standardized, validated system for defining the extent of the prolapse. The POP-Q points are converted to a staging system developed by the ICS [9]. The primary endpoint was clinical data at 6 months, assessed by the PFDI-SF20 Questionnaires, PISQ-12 Questionnaires, Patient-Phone Questionnaires, and Physician Satisfaction Questionnaires, in addition to analysis of the collected clinical data. Secondary end points were clinical data, PFDI-SF20 Questionnaires, and PISQ-12 Questionnaires at 12 months of follow-up.

### Xenform Matrix Placement

Xenform Matrix was soaked in sterile, room temperature 0.9% saline for approximately one minute, based on previously studied tissue characteristics, to accomplish the recommended hydration. The protocol intentionally did not define a specific procedure to determine the size or shape of the implant. The study was instead designed to test this material as used by different surgeons using their individual techniques. The supplied 8 cm × 12 cm sheet of Xenform Matrix was trimmed to the desired size and shape either before or after the hydration process, as determined by the surgeon. The graft was immersed in sterile saline until ready for use and was then sutured in place using standard suturing techniques with bites in the graft material and host tissue. Typically, sutures were first anchored to the ligament of interest under direct visualization, and then attached to the edges of the graft, though the specific surgical technique and suture material used was determined by the individual surgeon.

### Subject Evaluation

During the baseline subject evaluation a medical history was taken, and physical and pelvic examinations performed. Follow-up evaluations were performed at 2 weeks, 6 weeks, 3 months, 6 months, and 1 year after surgery. The subjects completed at baseline and at each follow-up visit the PFDI-20 and PISQ-12 questionnaires. The occurrence of intra-operative complications and postoperative adverse events was documented.

### Statistical Analysis

Descriptive statistics for continuous variables consisted of the mean and standard deviation (SD). The significance of symptom score changes at 6 months and 1 year were determined by the t-test for paired data. The rate of clinical success was calculated as the percentage of subjects showing improvement from baseline of at least one prolapse stage. Exact binomial 95% confidence intervals (CI) around point estimate of success rate were also computed. Between group difference in success rate was evaluated with the Fisher exact test. All statistical analyses were performed using SAS for Windows version 8.2 or higher (SAS institute Inc., Cary, North Carolina, USA).

## Results

Forty-six subjects were enrolled in the study. Forty-five subjects underwent pelvic floor reconstruction with the Xenform Matrix. Forty-three subjects completed the study. Of the 3 subjects who did not complete the study, one withdrew her consent after screening but before her surgery and the other two dropped out 3 months and 6 months after their surgery.

The demographics of the subjects are presented in Table 1. Twenty-three subjects (51%) had a prior hysterectomy, 18 percent had a prior anterior wall repair, 2% had a prior vault suspension, and 13% had a prior posterior wall repair. The most common presenting defect was a cystocele (98%), but 84% also had a rectocele. Fourteen subjects (31%) underwent a concomitant hysterectomy at the time of her prolapse repair. Forty-three subjects (96%) underwent general anesthesia during their reconstruction and the other 2 (4%) had spinal anesthesia. The average procedure time was 153 minutes (SD 53.0). The average length of stay for the subjects was 1.8 days (SD 0.5).

**Table 1:** Demographics and Baseline Characteristics

	Mean (SD)
**Age**	60.2 (11.8)
**BMI**	28.7 (5.5)
**Parity**	2.9 (1.1)
	**N (%)**
	
**On Estrogen Replacement**	14 (31.1)
**Post Menopausal**	37 (82.2)
**Prior Hysterectomy**	23 (51.1)
**Prior Prolapse Surgery**	16 (35.5)

The anatomical outcomes are listed in Table 2. The improvement from surgery was evident at the primary endpoint of 6 months, which persisted at the 1 year follow-up. At 1 year, 88% of subjects had a successful outcome based upon improvement of their ICS stage. The mean Point Ba on the POP-Q exam was +1.7 cm at baseline and improved to -1.7 cm at 1 year. The mean Point Bp on the POP-Q exam was -0.2 at baseline and improved to -2.5 cm at 1 year. These findings are statistically significant. The total vaginal length did not change from the surgery, however the vaginal apical support (point C) significantly improved by 130% after surgery (from -3.0 to -6.9 cm). There was no statistically significant change from the 6 month follow-up to the 1 year follow-up (Ba, C and TVL), however, the change for Bp was significant (*P *= 0.002 for the change between 6 months and 1 year). Mean PFDI-20 scores improved by greater than 70% (*P *< 0.001) at 6 months and this improvement remained the same at 1 year (Table 3). The PISQ-12 scores improved from baseline (*P *= 0.023) at 6 months and then were maintained at the 1 year exam (Table 4). Two patients reported dyspareunia, unrelated to the graft.

**Table 2:** Anatomical Outcomes

POP-Q Points	Baseline (cm)	6 Months (cm)	1 Year (cm)	*P *value*
**Ba**	1.7	-1.9	-1.7	<0.001
**Bp**	-0.2	-2.8	-2.5	<.0001
**C**	-3.0	-7.0	-6.9	<.0001
**TVL**	8.9	8.4	8.7	NS

NS: not statistically significant; POP-Q: Pelvic Organ Prolapse Quantification System

*compared to baseline exam

**Table 3:** Pelvic Floor Distress Inventory (PFDI-20) Results

Item	Baseline	6 months	1 Year
**Mean**	124.6	35.4	34.0
**SD**	61.2	32.1	31.7
*P *value*		<0.001	<0.001

SD = Standard deviation

*compared to baseline score

**Table 4:** Pelvic Organ Prolapse Sexual Questionnaire (PISQ-12) Results

Item	Baseline	6 months	1 Year
**Mean**	31.6	34.9	34.7
**Standard Deviation**	7.1	5.3	5.9
*P *value*		0.023	0.023

*compared to baseline score

Three subjects experienced one serious adverse event each; one of the adverse events (constipation) was deemed by the study physician to be unrelated to Xenform. One subject had severe pyelonephritis resulting in dialysis. This subject had a previous history of pyelonephritis, sepsis, and acute renal failure. The third subject had a reported recurrent cystocele of moderate severity, possibly related to the device. No graft related erosions or pain lasting more than 30 days were reported. No subjects withdrew due to an adverse event.

## Discussion

This multicenter study is the first to investigate the use of Xenform^® ^Matrix in pelvic floor reconstruction among subjects with POP. This study shows that at 1 year subjects who underwent pelvic reconstruction using this acellular noncrosslinked xenograft derived from fetal bovine dermis had statistically significant improvement in both subjective and objective measurements. There were no erosions noted with this material and it appeared to be safe and efficacious in the treatment of POP.

The current study demonstrated an 88% success rate (defined as ICS stage improvement relative to baseline), with mean Ba and Bp points on the POP-Q exam of -1.7 cm and -2.5 cm, respectively, at 1 year and PFDI-20 and PISQ-12 scores of 34.0 ± 31.7 and 34.7 ± 5.9, respectively. By comparison, a study of a bovine pericardium collagen matrix implant demonstrated a success rate (defined as Ba of more than -1) of 85.7% at 1 year [10]. In a study of a porcine xenograft matrix (Pelvicol, CR Bard, Covington, GA, USA), the surgical success rate was 94%, defined as grade 0 or grade 1 [11]. Paraiso et al. 2006 [12] studied a porcine small intestine submucosa graft in 32 patients and found a success rate of 54% (defined as Bp ≤ -2) at 1 year with a PFDI-20 score of 34 ± 37 (n = 24) and a PISQ-12 score of 33 ± 8. An extensive analysis of comparative studies assessing synthetic and biologic graft use for the treatment of anterior and posterior POP has also been conducted by the Society of Gynecologic Surgeons [13,14], including several studies of porcine dermal grafts (Pelvicol) reporting failure rates of 7-36%, depending on definition, at 12-15 months post-surgery [15-18] and Vicryl mesh synthetic grafts (Ethicon, Somerville, NJ, USA) reporting failure rates of 0-36% at 12-15 months [17,19].

The study protocol did not mandate a particular procedure for placement of the graft material, in order to allow the material to be studied in a variety of ways at the discretion of the individual surgeon. If a particular surgical technique or graft size had been mandated, the results could only be applied under those conditions, rather than across the broad variety of techniques. To date, no one particular technique or graft size has been shown to be the ideal procedure. In this study, the four separate clinical settings were able to customize the graft to individual preference with favorable subjective and anatomical findings.

One unexpected outcome from this study was the finding that the results at 6 months were almost identical to the 1 year outcomes (both success and failures). This finding demonstrates that in this trial the 6 month data points acted as a direct correlation to the 1 year data points. If a subject with the Xenform Matrix has success (both objective and subjective) at 6 months post surgery then one may expect these same results to hold at 1 year. This also was found in subjects having no relief at 6 months. If this finding holds true for other research in pelvic floor reconstruction graft materials then it can perhaps help design further trials with these materials. If researchers knew that 6 months in pelvic floor research results were equivalent to 1 year results, then trials might be designed in a more efficient manner. But it is not clear whether the 6-month findings would be stable with other (particularly non-xenograft) materials or other non-tested surgical repair techniques.

This single-arm, non-randomized study is limited by the lack of a control arm and the relatively small number of patients. The results from this trial should be confirmed within a larger, double-blind study. In addition, studies with longer follow-up beyond 1 year will be needed.

## Conclusions

This prospective study provides evidence of the safety and effectiveness of Xenform Matrix with respect to restoration of normal anatomy, symptom relief, quality of life, and sexual function over a 1-year period.

## List of Abbreviations

POP: Pelvic Organ Prolapse; POP-Q: Pelvic Organ Prolapse Quantification; PFDI-20: Pelvic Floor Distress Inventory; PISQ-12: Pelvic Organ Prolapse Sexual Questionnaire

## Declaration of Competing Interests

Dr. Goldstein reports being a proctor for C.R. Bard, Inc. since July 2009. Drs. Patel and Maccarone report being proctors for Boston Scientific Corporation, Inc. Dr. Naughton reports owning stock in Caldera Medical, Inc. Dr. Aguirre reports receiving research funding and/or consultant honoraria from Boston Scientific Corporation, ETHICON Women's Health & Urology, American Medical Systems, Medtronic Inc, Synovis, and Boehringer Ingelheim Pharmaceuticals, Inc.

## Authors' contributions

All authors participated in the design and conduct of the study. HBG drafted the manuscript. All authors reviewed and approved the final version of the manuscript.

## Pre-publication history

The pre-publication history for this paper can be accessed here:

http://www.biomedcentral.com/1471-2490/10/21/prepub
